# Body, Space, and Pain

**DOI:** 10.3389/fnhum.2014.00369

**Published:** 2014-05-28

**Authors:** Jörg Trojan, Martin Diers, Camila Valenzuela-Moguillansky, Diana M. E. Torta

**Affiliations:** ^1^Department of Psychology, University of Koblenz-Landau, Landau, Germany; ^2^Department of Cognitive and Clinical Neuroscience, Central Institute of Mental Health, Medical Faculty Mannheim / Heidelberg University, Mannheim, Germany; ^3^Institute of Complex Systems of Valparaíso, Valparaíso, Chile; ^4^Department of Psychology, University of Turin, Turin, Italy

**Keywords:** body perception, pain, psychology, neuroscience, spatial attention

## Theoretical Background

Chronic pain is accompanied by a variety of alterations in body perception. Pain patients often exhibit distortions in the perception of limb positions and sizes: back pain patients have problems in delineating the outline of their backs and their body image is distorted in the painful area (Moseley, 2008). Patients with Complex Regional Pain Syndrome (CRPS) suffer from intense pain in their affected hand and often perceive it as being larger than it actually is (Moseley, 2005). Amputees often report pain in their amputated limb and the amount of pain seems to be related to distorted spatial perception of the limb (Grüsser et al., 2001). Results from CRPS patients show that not only the perception of the body is affected, but that pain leads to a distorted perception of the peripersonal space surrounding the body (Reinersmann et al., 2011). At a more fundamental level, sensory changes associated with chronic pain states cannot be explained by peripheral deficits alone, rather, cortical representations seem to be involved as well.

Research on how body perception and pain are linked to each other has been conducted from different and possibly overlapping perspectives. One line of research highlights the importance of attentional biases toward or away from the location of pain. Another approach underlines the role of multisensory representation of our body and the surrounding space in the perception of pain and nociceptive stimuli. Extending this latter view, there is also work focusing in particular on the complex interplay between motor action and pain perception.

Experimental studies on the relationship between body perception and pain are contributing to our understanding of clinical findings and the development of new treatment approaches: nociceptive stimuli draw attention to their locations (Van Damme et al., 2007) and pain stimuli are perceived as less intense if attention is drawn away from their location (Van Ryckeghem et al., 2011). Vision of stimulated body parts reduces pain perception (Longo et al., 2009; Diers et al., 2013) and the larger the perceived size of a limb, the stronger is the effect (Mancini et al., 2011). Ongoing nociceptive input alters somatosensory localization (Trojan et al., 2009) and conflicts between frames of reference onto which somatosensory stimuli are localized modulate the perception of the stimuli (Gallace et al., 2011; Torta et al., 2013). Studies about the relationship between somatosensory inputs and motor planning suggest that visual–motor feedback leads to “peculiar” (and sometimes painful) sensations (McCabe et al., 2005).

## Overview of the Articles in This Research Topic

In this Research Topic, three studies focused on the effect of painful or nociceptive stimulation on body representation and postural control. Bouffard et al. (2013) applied pain to the right arms of healthy participants and observed that the subjective body midline shifted to the right; non-painful vibration applied to the left arm also led to a right-shift. The pain-specific shift toward the stimulated side might be functionally beneficial to protect the painful area of the body. Steenbergen and collaborators assessed the agreement of spatial perceptual maps for touch and nociception and proposed that a common internal body representation can underlie spatial perception of touch and nociception (Steenbergen et al., 2012). Lelard and collaborators showed that imagining being in a painful situation induces changes in postural control and leg muscle activation, thus resulting in an increased stiffness compared to non-painful situations (Lelard et al., 2013). Anelli and colleagues did not study the effect of pain directly but explored the effect that potentially dangerous approaching stimuli have on motor responses: static neutral objects triggered faster responses compared to static dangerous ones (Anelli et al., 2013). Responses to dynamic objects were instead affected by the direction of the movement of the object.

Two studies addressed the question of how changes in body representations affect pain processing. In a virtual reality study, Martini and colleagues demonstrated that pain thresholds could be top-down modulated by changing the color of a seen arm (Martini et al., 2013). Pia and collaborators showed that right-brain damaged patients could feel pain in response to a stimulus produced in a foreign arm that was subjectively experienced as their own (Pia et al., 2013).

The interplay between movement, sensory feedback, and pain was specifically addressed by two studies. Foell and collaborators used a mirror setup in order to clarify the impact of sensory motor incongruence on pain perception (Foell et al., 2013). The results revealed that sensorimotor conflict affected perceived body integrity but was not sufficient to trigger substantial pain experiences in healthy volunteers. Meulders and colleagues induced pain-related fear of movements in healthy participants and demonstrated that this fear could be generalized to similar movements (Meulders et al., 2013). These findings may yield a better understanding of the role of fear and avoidance in chronic musculoskeletal pain.

Four studies were conducted on clinical populations. Preißler et al. (2013) looked for the influence of prosthesis use in upper limb amputees on phantom limb pain and cortical thickness and suggested a relationship between prosthesis use and cortical plasticity of the visual stream. This plasticity might present a brain adaptation process to new movements and coordination patterns needed to guide an artificial hand. Riquelme and colleagues showed that administration of a somatosensory therapy could reduce pressure pain sensitivity in adults with cerebral palsy, without affecting other abilities such as texture recognition or tactile sensitivity (Riquelme et al., 2013). Turton and colleagues presented a new method to report body perception disturbances, targeted at patients with CRPS (Turton et al., 2013). Patients could manipulate a variety of features of an avatar on a computer screen, yielding 3-D representations of their symptoms. Wallwork and colleagues aimed at dissociating the ability to judge the rotation of the neck as compared to the rotation of the hand in dizzy people and observed that those patients show generally slower responses independently of to the task (Wallwork et al., 2013).

Finally, broadening the focus to social neurosciences, Krahé and collaborators proposed three factors playing a major role in pain perception: (1) the relationship between the person in pain and the social partner; (2) the possibility of the person in pain to understand the social partner’s intentions; and (3) the degree to which the person in pain sees the social partner’s possibility to act (Krahé et al., 2013).

## Conclusion

The findings presented here demonstrate that the subjective experience of pain can only be understood in a larger framework of body representations and peripersonal space. The relationship between pain and these spatial representations is bidirectional, but the underlying neurophysiological mechanisms are not yet known. Regardless of this present lack of knowledge, the results reported in this Research Topic clearly bear clinical significance: future pain diagnostics and treatment approaches will benefit from putting more stress on body perception.

## Conflict of Interest Statement

The authors declare that the research was conducted in the absence of any commercial or financial relationships that could be construed as a potential conflict of interest.
